# Large-N Rat Data Enables Phenotyping of Risky Decision-Making: A Retrospective Analysis of Brain Injury on the Rodent Gambling Task

**DOI:** 10.3389/fnbeh.2022.837654

**Published:** 2022-04-25

**Authors:** Cole Vonder Haar, Michelle A. Frankot, A. Matthew Reck, Virginia Milleson, Kris M. Martens

**Affiliations:** ^1^Department of Psychology, West Virginia University, Morgantown, WV, United States; ^2^Department of Neuroscience, Ohio State University, Columbus, OH, United States

**Keywords:** Iowa Gambling Task (IGT), impulsivity, controlled cortical impact (CCI), statistical approaches, rat

## Abstract

Decision-making is substantially altered after brain injuries. Patients and rats with brain injury are more likely to make suboptimal, and sometimes risky choices. Such changes in decision-making may arise from alterations in how sensitive individuals are to outcomes. To assess this, we compiled and harmonized a large dataset from four studies of TBI, each of which evaluated behavior on the Rodent Gambling Task (RGT). We then determined whether the following were altered: (1) sensitivity to overall contingencies, (2) sensitivity to immediate outcomes, or (3) general choice phenotypes. Overall sensitivity was evaluated using the matching law, immediate sensitivity by looking at the probability of switching choices given a win or loss, and choice phenotypes by k-means clustering. We found significant reductions in sensitivity to the overall outcomes and a bias toward riskier alternatives in TBI rats. However, the substantial individual variability led to poor overall fits in matching analyses. We also found that TBI caused a significant reduction in the tendency to repeatedly choose a given option, but no difference in win- or loss-specific sensitivity. Finally, clustering revealed 5 distinct decision-making phenotypes and TBI reduced membership in the “optimal” type. The current findings support a hypothesis that TBI reduces sensitivity to contingencies. However, in the case of tasks such as the RGT, this is not a simple shift to indiscriminate or less discriminate responding. Rather, TBI rats are more likely to develop suboptimal preferences and frequently switch choices. Treatments will have to consider how this behavior might be corrected.

## Introduction

Traumatic brain injury (TBI) affects 2.8 million Americans every year and is associated with impairments in decision-making (Bhalerao et al., 2013; Zgaljardic et al., 2015). Though these psychiatric-like symptoms are well-cataloged in this population, the underlying behavioral and neurological mechanisms are not clear. A better understanding of the behaviors that lead to these symptoms may yield effective rehabilitative strategies which could readily be implemented. Moreover, study of this population may lead to additional insights regarding the fundamentals of behavior. Patients with TBI display altered performance of numerous neuropsychological assessments related to decision-making, however, as described below, findings are not necessarily in line with a simple hypothesis of “injury increases risk taking.” Shifts in behavior such as reduced sensitivity to outcomes or reduced learning rates may also be sufficient to explain such findings.

In the Iowa Gambling Task (IGT), which evaluates preference for safe vs. risky alternatives as people interact with the choices, patients with TBI make increased risky decisions (Sigurdardottir et al., 2010; Cotrena et al., 2014; Visser-Keizer et al., 2016). On the Game of Dice Task, which explicitly presents probabilities in the form of dice, patients with TBI made significantly fewer advantageous (safer) choices (Rzezak et al., 2012). However, in the Balloon Analog Risk Task, which presents a visual representation of risk/reward in the form of a virtual “balloon” that participants “inflate” to earn points, patients with TBI demonstrate no differences (in adolescents, Chiu et al., 2012), or even risk aversion (in adults, Fecteau et al., 2013). While the IGT is the most widely used of these tasks, a large confound is that outcomes must be learned over time through interaction. However, this also likely yields the best translational value as explicit consequences of an action are rarely specified in real life at the time of a decision. In contrast, the Game of Dice Task gives indicators of probability, but some level of math equivalency must be carried out (e.g., 4/6 numbers on a die = 0.67 probability for $100, weighed by cost of bet). Finally, the Balloon Analog Risk Task likely provides the simplest representation of risk in the form of a (virtual) balloon which inflates to a point of popping. However, even with this task, some level of learning is required to determine the elasticity of the balloon and maximize gains. In the case of the Fecteau study, the “risk aversion” observed in patients with TBI was largely due to a lack of adaptation. This suggests more general deficits of learning over time as opposed to a fundamental change in preferences regarding risk.

While TBI clearly alters decision-making, given the somewhat discrepant findings and the nature of the assessments, it is difficult to conclude that injury explicitly increases risky decision-making. Instead, insensitivity to outcomes (e.g., a winning or losing trial) or reduced learning from those outcomes over time may also account for these same symptoms. Indeed, earlier work explicitly tested this in patients with TBI and found that they had difficulty discriminating outcomes and adjusting their own actions based on those outcomes (Schlund and Pace, 2000; Schlund, 2002). To evaluate these findings with greater control, animal models may be used. With the appropriate motivation, animals can be trained on an array of behaviors similar to the human condition. In a rat model of TBI, we have reported findings strikingly similar to the human condition. Rats with either a frontal or unilateral TBI demonstrated reduced optimal decision-making on an analog of the IGT, the Rodent Gambling Task (RGT) (Shaver et al., 2019; Ozga-Hess et al., 2020), in which rats can make safe or risky choices by nosepoking in different holes in an operant chamber. Moreover, they distributed their choices to both a less risky (but suboptimal) choice, and riskier choices. This suggests a more indiscriminate style of decision-making as opposed to a pure increase in riskiness. Indeed, in studies of simple discrimination after TBI in rats, impairments are substantial (Martens et al., 2012; Vonder Haar et al., 2014; Muelbl et al., 2018). However, while these deficits resolve, more complex decision-making tasks such as the RGT may present less explicit feedback than discrimination tasks and present a much greater challenge to detection of contingencies.

A large drawback to the existing studies described above are the relatively small samples sizes. With heterogenous behaviors such as decision-making, individual differences may make it difficult to determine whether patients or animals are truly more risk-preferring or if there have merely been reductions in sensitivity to outcomes. The use of larger scale, harmonized datasets may provide an opportunity to gain insight and evaluate different potential explanations for changes in choice behavior. In the field of behavioral science, two theoretical approaches are commonly used to describe many types of decision-making. The molar viewpoint takes the perspective that behavior is sensitive to the overall rates of reinforcement amongst alternatives (Baum, 1989). In contrast, the molecular viewpoint suggests that immediate outcomes drive subsequent decisions (Shimp, 2020). Indeed, experimental setups can be designed which provide evidence for both, but some combination of the two are likely at work for everyday behaviors. The molar view is epitomized by the Matching Law, a mathematical description that relative rates of behavior closely match relative rates of reinforcement (Baum, 1974). The strongest evidence for this comes from data collected at a steady state, after the contingencies have been learned, using experimental setups in which effort is independent of time spent on an alternative (i.e., interval schedules as opposed to ratio schedules) (Rider, 1981). The strongest evidence for molecular viewpoints comes from tasks in which change occurs rapidly or frequently and behavior must be adapted to new contingencies (Dalton et al., 2014). Because changes to either molar or molecular sensitivities after TBI could drive changes to decision-making, both must be evaluated. Moreover, if neither are sufficient to describe behavior at the group and/or subject level, atheoretical statistical data reduction techniques may provide insight into these changes underlying changes to decision-making.

In the current set of analyses, we used a dataset collected across four studies to evaluate molar (overall contingencies), molecular (immediate contingencies), and atheoretical accounts (purely descriptive) of behavior. This large dataset was able to power analyses which would have been impossible with data from any single one of these studies. The RGT captures decisions across four distinct alternatives, each associated with a different probability and magnitude of reinforcement (sucrose pellets) and punishment (time out from reinforcement). Because choices are mutually exclusive and probabilistic, decisions should collapse into exclusive preference of the most optimal option. However, this is rarely observed at a subject level, and never at the population level. Thus, there is rationale to evaluate if behavior is allocated according to relative rates of reinforcement (i.e., matching: molar sensitivity) or if there are high sensitivities to immediate outcomes (i.e., shifting behavior based on a “win” or “loss”: molecular sensitivity). TBI causes changes to decision-making behavior on this task (Shaver et al., 2019; Ozga-Hess et al., 2020) and may disrupt sensitivity to either molar or molecular outcome dynamics. In the current study, we aimed to compare molar, molecular, and atheoretical accounts of RGT choice behavior.

## Materials and Methods

### The Dataset: Rodent Gambling Task Performance in a Large Cohort of Traumatic Brain Injury and Sham Rats

The dataset analyzed in the current study was compiled from four separate experiments (Table 1). Common methods are described below in brief. All rats performed the Rodent Gambling Task, a measure of probabilistic decision-making and motor impulsivity. The first study assessed the effects of a bilateral frontal TBI delivered either before (“acquisition” condition), or after (“trained” condition), learning the RGT (Shaver et al., 2019). The second assessed the effects of a unilateral TBI on acquisition of RGT learning (Ozga-Hess et al., 2020). The remaining two are in preparation for publication, but both used bilateral frontal TBI. One assessed the effects of a dietary manipulation before injury (RGT trained pre-injury), and the other a drug treatment after injury (RGT tested in acquisition). For these two studies, the control conditions of sham surgery or TBI surgery (no additional treatment/manipulation) were isolated for the current analysis. For all experiments, stable performance was evaluated (i.e., sessions ≥ 15 for pre-injury and sessions > 10 for post-surgery) to mitigate learning effects in acquisition experiments. Thus, the sessions selected represent approximately weeks 4–8 post-injury. To maximize control numbers, both pre-TBI and post-surgery sham data were pooled for any Sham-only analyses (e.g., single-subject plots). This resulted in 80 Sham animals, and 51 TBI animals with an average of 17 sessions each. Analyses comparing Sham and TBI performance were carried out using only post-injury data (Sham = 58, TBI = 51). Three types of analysis (representing molar, molecular, and atheoretical perspectives) were evaluated on this dataset to better understand (1) normal probabilistic decision-making, and (2) how this was disrupted by brain injury.

**TABLE 1:** Brief description of studies.

Study	References	Injury	Trained or acquisition	N (TBI)
1	Shaver et al., 2019	Bilateral frontal	Both (separate cohorts)	44 (21)
2	Ozga-Hess et al., 2020	Unilateral parietal	Acquisition	25 (11)
3	Under review	Bilateral frontal	Trained	18 (8)
4	In preparation	Bilateral frontal	Acquisition	22 (11)

### Subjects

Subjects were 109 male Long-Evans rats, between 3 and 5 months of age at time of injury. Rats were either pair-housed in standard cages (Allentown, Allentown NJ) or triple-housed in larger, pentagonal cages (Animal Care Systems, Centennial CO) prior to injury and single-housed after injury. Rats were restricted to 12–14 g of chow daily plus pellets earned during the task. Water was available *ad libitum*.

### Apparatus

Behavioral testing was conducted in a set of 16 standard 5-choice operant chambers (Med Associates, St Albans, VT). Each was enclosed in a sound-attenuating box, and white noise played in the room. The right side was equipped with a food hopper and light. The left wall of the chamber was equipped with a 5-hole array in which rats’ nosepokes were recorded. The chamber was also equipped with a houselight.

### The Rodent Gambling Task

Rats were trained as previously reported on the RGT (Zeeb and Winstanley, 2013; Shaver et al., 2019; Ozga-Hess et al., 2020). In brief, nosepoking behavior was shaped by reinforcing pokes to an illuminated hole. The stimulus duration was gradually decreased until rats responded within 10 s and responses made prior to illumination were recorded as “premature” and punished with a timeout. Rats then began “forced-choice” RGT training in which only one option was available, but the RGT contingencies were in effect. Following 7 sessions of forced choice, rats were tested on the free-choice RGT.

The choice contingencies on the RGT are designed such that four options are available, named for the number of pellets they deliver: P1 (90% 1 pellet; 10% 5-s timeout), P2 (80% 2 pellets; 20% 10-s timeout), P3 (60% 3 pellets; 40% 30-s timeout), and P4 (40% 4 pellets; 60% 40-s timeout). The P2 option is optimal (13.71 pellets/min), the P1 suboptimal, but low risk (9.81 pellets/min), while the P3 (4.5 pellets/min) and P4 (3.31 pellets/min; least advantageous outcome) are high risk but with large magnitudes.

Multiple other variables were collected on the RGT, including the number of premature/impulsive responses, omitted trials, total trials, total reinforcers, response latency, collection latency, and perseverative pokes to the 5-choice array.

### Traumatic Brain Injury: Controlled Cortical Impact

A controlled cortical impact procedure was used to administer moderate-severe, focal TBI (Hoffman et al., 1994). Rats were anesthetized with isoflurane (5% induction, 2–4% maintenance) in 0.5 L/min oxygen. Then rats were placed into a stereotaxic frame and administered a local analgesic (Bupivacaine; 0.25%, s.c.) at the incision site and a subcutaneous general analgesic (ketoprofen; 5 mg/kg, s.c.). Then, the surgical site was sanitized, and rats were given a midline incision. A craniectomy was performed above the injury location and a controlled cortical impact delivered (bilateral frontal: + 3 mm/ + 0 mm/−2.5 mm @ 3 m/s; unilateral parietal: −2.4 mm/ + 2.4 mm/−2.5 mm @ 3 m/s). Sham surgeries consisted of either craniectomy shams (all procedures except impact) or “intact” shams which only received an incision. Rats resumed testing starting at week 2 post-injury and continued until week 8–12 (varied by study). Sessions evaluated here would represent approximately weeks 4–8 post-injury, a relatively chronic time point for rats.

### Data Processing

Raw trial-by-trial data were imported into R for processing. Any manipulations/treatments other than TBI were filtered away. Sessions prior to stable post-injury performance were filtered away. For each subject, session and choice option, total choices, earned pellets, and total timeout were summed. Additional calculations were performed as described below.

### Experiment 1—Molar Accounts of Behavior: The Generalized Matching Law

To maximize pellets earned, the P2 option should be chosen exclusively. However, this is not observed at the population level (even in Sham rats) and rarely observed in individual rats. To determine if this heterogeneity in choice performance was related to relative reinforcement rates amongst the choice options, the generalized matching law was evaluated. The matching law stipulates that behavioral allocation will approximate relative reinforcement rate (Baum, 1974). While this typically breaks down under ratio schedules (probabilistic delivery in this task is analogous to variable ratio), it may account for some behavior.

The ratio of choices for each option was calculated per subject and for the last 10 sessions of performance. The obtained reinforcers and punishers were used to calculate a reinforcement rate for each option. When total choices were less than 4, the programmed reinforcement rate was used. When choice of an option was 0, this was replaced with a 1 and added to the total of choices. These adjustments were necessary to prevent 0 s and infinite values since this experiment was not designed to eliminate those from the data as would be typical in a matching experiment. The ratios of choice and reinforcement rate [e.g., P1/(P2 + P3 + P4)] were then calculated for each option, subject, and session. These were log transformed and fit to the generalized matching law using linear regression [log(R_*T*_/R_*O*_) = a*log(B_*T*_/B_*O*_) + log(b), where R_*T*_ is the reinforcement rate of the target option and R_*O*_ is the sum of reinforcement rate of other options, B_*T*_ is the number of choices on the target option, B_*O*_ is the sum of choices on other options, and b is the bias].

From this linear regression, values representing the sensitivity to reinforcement (slope), bias (y-intercept) and the overall fit (*R*^2^) were calculated. Plots of individual subjects were used to visualize how many rats demonstrated matching, and a quantitative comparison (*t*-test) was made between TBI and Sham. Average values were used to also fit the matching law at the population level to Sham and TBI.

### Experiment 2—Molecular Accounts of Behavior: Win/Loss Sensitivity

It is possible that in tasks such as these, greater sensitivity is given to immediate consequences. Thus, if a choice is reinforced on a given trial, this may increase what is commonly referred to as “win-stay” behavior, or an increase in probability of staying on that option. Conversely, punishment may increase “lose-shift” behaviors, or the probability of switching away from that option. To maximize performance on the RGT, rats must persist through punishment on optimal choices (i.e., P2 option) and switch away from reinforcement on riskier choices (e.g., P3, P4).

The probability of staying with the same choice on a subsequent trial was evaluated as a function of the preceding outcome (“win” or “loss”). Overall data were calculated for the last 10 sessions from a given study. To power analyses at the individual choice option level, data from the last 10 sessions for each choice option were summed to a single value and filtered so that only choices with greater than 4 observations were used. Overall switching, probability given a win, and probability given a loss were evaluated in a linear mixed-effects regression with Injury and Session as predictors. Individual subjects were plotted to visually examine the range of sensitivity between the two groups. The same analyses were then carried out for each choice option (i.e., P1:P4) but using ANOVA since data were aggregated.

### Experiment 3—Atheoretical Accounts of Behavior: k-Means Clustering

Given that neither opposing theories of behavior strongly accounted for RGT data, a third experiment was conducted to determine if an unbiased approach might better describe the data. A simple k-means clustering approach was used. In this, the distance from a multidimensional centroid was minimized by categorizing subjects into *k* number of clusters. Data were averaged on a per subject basis from the last 10 sessions of a given study. A series of clusters was evaluated, starting at 2, and increasing to 10. Clusters were evaluated using the gap statistic to determine optimal number and validated by visual inspection of the elbow plot of the sum of squares. To reduce risk of overfitting/overestimation given the relatively small dataset, the number of subjects in each cluster were also monitored to ensure that any given cluster contained at least 5% of the sample. Sham data were assessed alone first, followed by TBI alone, and then the full dataset together. Visualization of the cluster averages were used to generate descriptive names as “phenotypes.”

### Supplemental Analyses

The supplement provides several comparisons between the various sub-group variables in the current study. Specifically, comparisons were made between craniectomy and intact shams, bilateral frontal and unilateral parietal TBI, and within-subjects pre- and post-injury effects. These subgroup comparisons (with the exception of intact vs. craniectomy) are of lower power than the main document, and so should be interpreted with caution.

## Results

### General Rodent Gambling Task Performance and Effects of Traumatic Brain Injury

**FIGURE 1:**
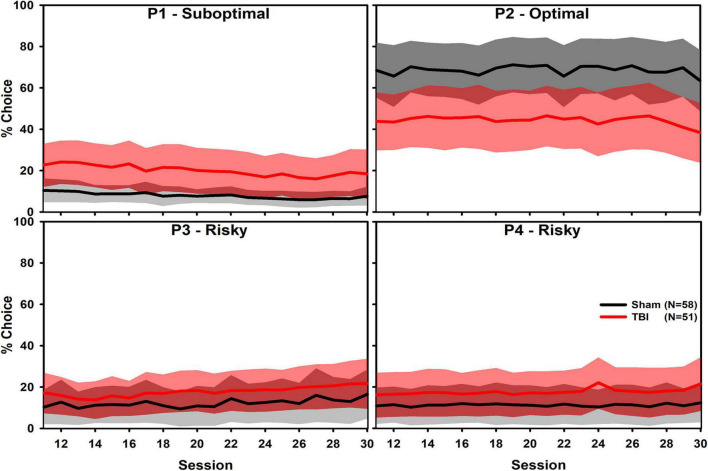
Overall choice of the four RGT options shown as mean (solid line) and one standard deviation band (fill). Only sessions after 10 are shown to maximize visualization of stable choice preference, which represents approximately weeks 4–8 post-injury. There were significant Injury effects on each choice option (P1 and P2: *p* < 0.001; P3: *p* = 0.048; P4: *p* = 0.022) and P1 had a slight, but significant decline over time (*p* = 0.001). Analyses indicated substantial individual variation in choice.

An examination of the relation between non-choice and other variables is presented in Supplementary Figure 1. There were substantial correlations between overall choice and multiple variables of interest, including premature responses and “Win-Stay” and “Lose-Shift” behaviors. Choice of each option was analyzed in independent linear mixed effects regressions, interacting Injury and Session as fixed effects, and allowing slope and intercepts to vary by subject as random effects. Sessions from 11 to 30 were selected to minimize early change after TBI and learning (Figure 1). For P1 choice, there were significant increases due to Injury and across Session (β = 0.68, *t* = 4.25, *p* < 0.001; β = −0.10, *t* = 4.00, *p* < 0.001), but not with regard to their interaction (β = −0.05, *t* = 1.26, *p* = 0.209). Individual subjects also varied considerably in their intercept but not in slope (SD = 1.04; SD = 0.03). For P2 choice, there was a significant decrease due to Injury (β = −0.79, *t* = 4.76, *p* < 0.001), but no effect of Session nor their interaction (β = 0.02, *t* = 0.80, *p* = 0.427; β = 0.01, *t* = 0.22, *p* = 0.825). Individual subjects also varied considerably in their intercept but not in slope (SD = 0.98; SD = 0.03). For P3 choice, there was a significant increase due to Injury (β = 0.35, *t* = 2.00, *p* = 0.048), but not because of Session nor their interaction (β = 0.03, *t* = 1.07, *p* = 0.288; β = 0.01, *t* = 0.17, *p* = 0.863). Individual subjects also varied considerably in their intercept but not in slope (SD = 0.86; SD = 0.03). For P4 choice, there was a significant increase due to Injury (β = 0.41, *t* = 2.32, *p* = 0.022), but no effect of Session nor their interaction (β = −0.01, *t* = 0.30, *p* = 0.767; β = 0.02, *t* = 0.63, *p* = 0.529). Individual subjects also varied considerably in their intercept but not in slope (SD = 1.03; SD = 0.03).

Although the P2 option had a large TBI effect, others were considerably smaller and likely only significant due to the large power given the number of subjects. Moreover, there was substantial individual variability as shown by the standard deviation of the random effects. This variability is described further in individual subject-level plots below. The magnitude of these individual differences were of similar or larger magnitude than the group-level effect. These reinforce the need to examine data on an individual subject level in subsequent analyses.

### Experiment 1: Molar Accounts of Behavior

**FIGURE 2:**
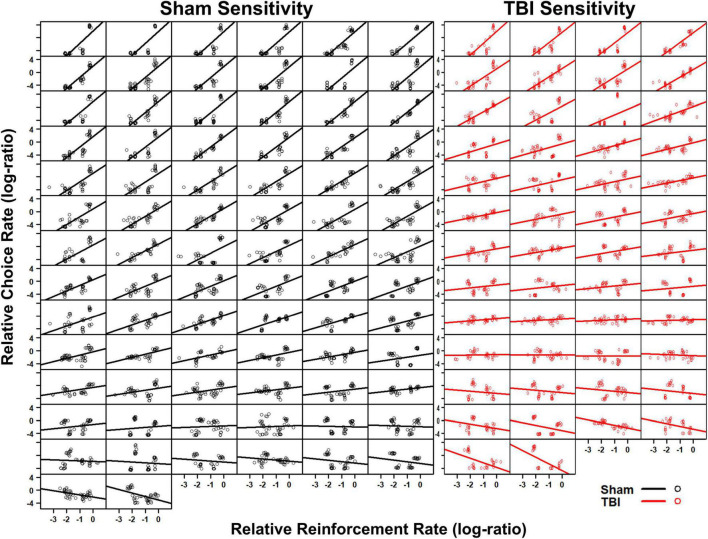
Individual subject fits to the matching law show Sham (left, black) and TBI (right, red) subjects. A wide degree of sensitivity was present across subjects. The matching law only described a very small subset of animals.

**FIGURE 3:**
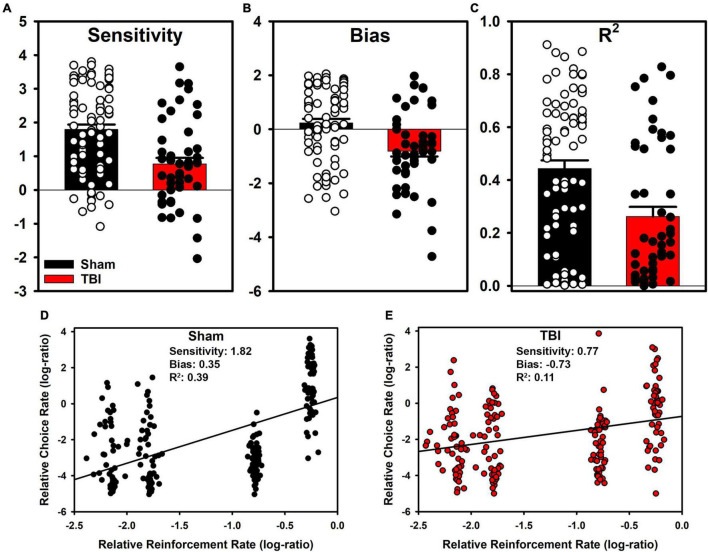
Aggregate matching law parameters and group-level fits. **(A)** TBI reduced sensitivity (*p* < 0.001), and **(B)** shifted bias toward suboptimal options (*p* < 0.001). **(C)** The matching law described TBI rats more poorly than Sham (*p* < 0.001), however the matching law did not describe data well at the individual or **(D,E)** at the aggregate level.

The generalized matching law was fit to each individual subject. In Sham rats, a large number were sensitive to the reinforcement contingencies as indicated by steep slopes in Figure 2. However, a portion also demonstrated anti-matching or preference for the riskier option as well as indifference to the relative rates of reinforcement. For the TBI rats, similar styles were present at the individual level (Figure 2). However, in the aggregate, TBI rats had reduced sensitivity to reinforcement rates [*t*_(107_._21)_ = 4.15, *p* < 0.001], increased bias toward risky alternatives [*t*_(104_._02)_ = 3.96, *p* < 0.001], and worse fits to the equation [*t*_(107_._25)_ = 3.64, *p* < 0.001] relative to Sham rats (Figures 3A–C). Further, the matching law fit poorly at the population level (Sham *R*^2^ = 0.39, TBI *R*^2^ = 0.11; Figures 3D,E).

### Experiment 2: Molecular Accounts of Behavior

**FIGURE 4:**
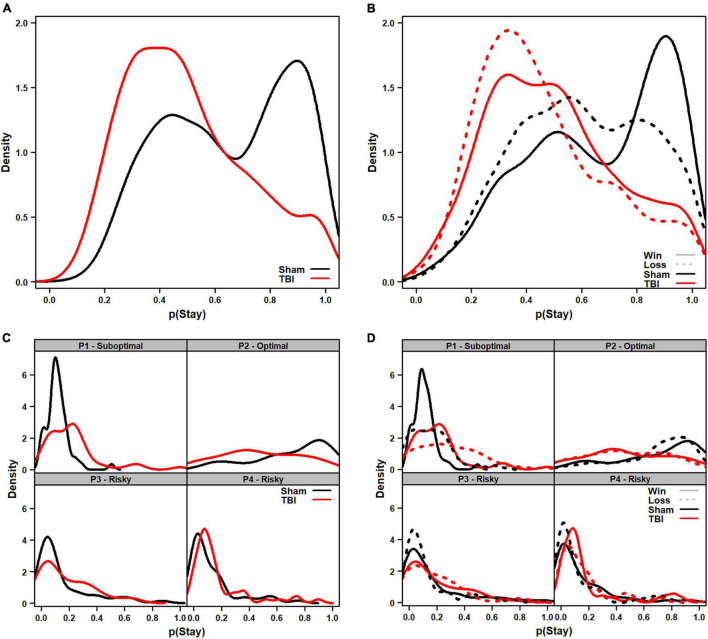
Density plots of distributions of the probability of staying with a choice in TBI vs. Sham animals. **(A)** Overall tendency to stay on a choice (regardless of outcome) was higher in Sham than TBI rats (*p* = 0.008). Sham rats displayed a bimodal set of peaks around 40 and 90% likelihood of staying on an outcome. **(B)** Breakdown of tendency to stay depending on whether the prior outcome was a win (solid) or loss (dashed). The same overall differences were present in TBI (*p* = 0.008), and losses reduced the probability of staying (leftward shift in curve; *p* = 0.016) but TBI rats did not show differential sensitivity to wins or losses (*p* = 0.966). **(C)** When broken down to each choice option, TBI rats were more likely to stay with P1 (*p* = 0.001), but less likely to stay with P2 (*p* < 0.001) regardless of outcome. **(D)** When choice option data were analyzed depending on whether prior outcome was a win (solid) or loss (dashed), similar overall effects in tendency to stay were observed with TBI rats more likely to stay with P1 (*p* < 0.001) and P4 (*p* = 0.021), but less likely for P2 (*p* < 0.001). There were no differential effects in sensitivity to wins vs. losses (*p*’s > 0.134).

To determine if immediate outcomes influenced decision-making on the RGT, likelihood of switching after a choice was analyzed, including session as a covariate. Aggregate distributions of switching are shown as density plots in Figures 4A,B. The overall tendency to stay was significantly reduced in TBI rats [*F*_(1_, _155_._25)_ = 7.07, *p* = 0.009]. When analyzed by the prior trial being a win or loss, TBI rats still were significantly less likely to stay with an option regardless of win or loss [*F*_(1_, _153_._1)_ = 7.29, *p* = 0.008], and overall rats were less likely to stay following a loss [*F*_(1, 3710)_ = 5.85, *p* = 0.016], but there was no differential sensitivity to losses in the TBI group [*F*_(1, 3710)_ = 0.00, *p* = 0.966].

While useful to capture global changes in propensity to stay with a choice, analyzing the overall data could miss important differences related to individual choice contingencies. Thus, a similar analysis was conducted, but data from the 10 sessions were summed for each option to ensure sufficient resolution. Aggregate distributions are shown as density plots in Figures 4C,D. For overall tendency to switch, there was an interaction of Injury and Choice Option [*F*_(3_, _407)_ = 12.74, *p* < 0.001], so each was analyzed separately. TBI rats were significantly more likely to stay on P1, but less likely on P2 choices [*F*_(1_, _105)_ = 11.44, *p* = 0.001; *F*_(1_, _106)_ = 19.43, *p* < 0.001], but not P3 or P4 [*F*_(1_, _99)_ = 0.75, *p* = 0.388; *F*_(1_, _97)_ = 2.32, *p* = 0.131]. When analyzed by win- and loss-trials, there was also an Injury × Choice Option interaction [*F*_(3_, _812)_ = 23.28, *p* < 0.001], so each was analyzed separately. TBI rats were significantly more likely to stay on P1 and P4 choice options, but less likely on P2 [*F*_(1_, _208)_ = 20.04, *p* < 0.001; *F*_(1_, _194)_ = 5.41, *p* = 0.021; *F*_(1_, _212)_ = 38.08, *p* < 0.001], and not significantly different on P3 [*F*_(1_, _198)_ = 1.37, *p* = 0.243]. There was no injury-related difference in tendency to switch given a win vs. a loss (*p*’s > 0.134).

**FIGURE 5:**
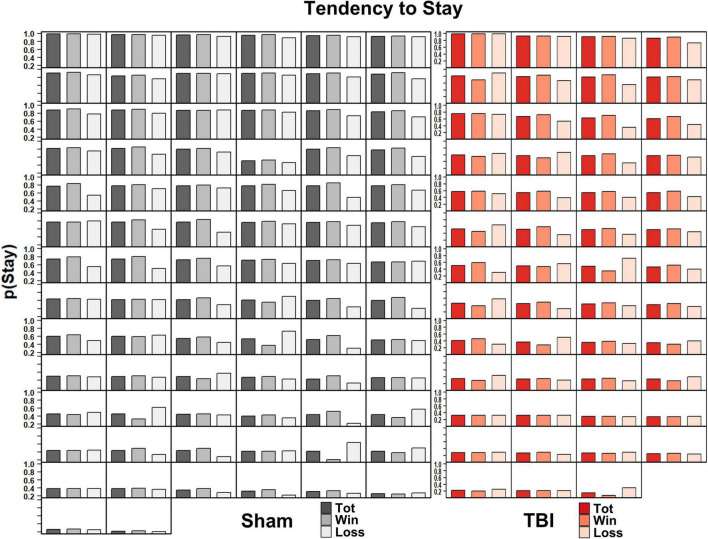
Individual subject’s probability of staying with a choice across total trials, winning trials, and losing trials in Sham (left, black) and TBI (right, red) subjects. There was considerable variability in the tendency to stay with a choice across subjects.

These wide distributions suggest considerable individual variability. Indeed, this was confirmed from viewing the average probability of staying with an option at the subject level (Figure 5). Both Sham and TBI groups had some rats which exploited options, and others which were frequently switching amongst choices.

### Experiment 3: Atheoretical Accounts of Behavior

For the Sham cohort, four clusters were the optimal fit to the data according to the gap statistic, and it was not until six clusters that any given one approached the < 5% sample threshold we established. For the TBI cohort, nine clusters was the optimal fit according to the gap statistic. However, examination showed that eight or more clusters resulted in clusters with less than 5% of the sample. A re-analysis of the TBI cohort, limited to a max of seven clusters identified seven as optimal on the gap statistic.

Once the data were combined, the gap statistic identified four clusters as optimal, and six or more clusters resulted in at least one with < 5% of a given group. Because the k-means algorithm is agnostic to injury condition, when four clusters were examined, the group-level fits were imprecise. The cluster number was increased to five and group-level data fit the clusters much better while still staying within the previously set parameters.

**FIGURE 6:**
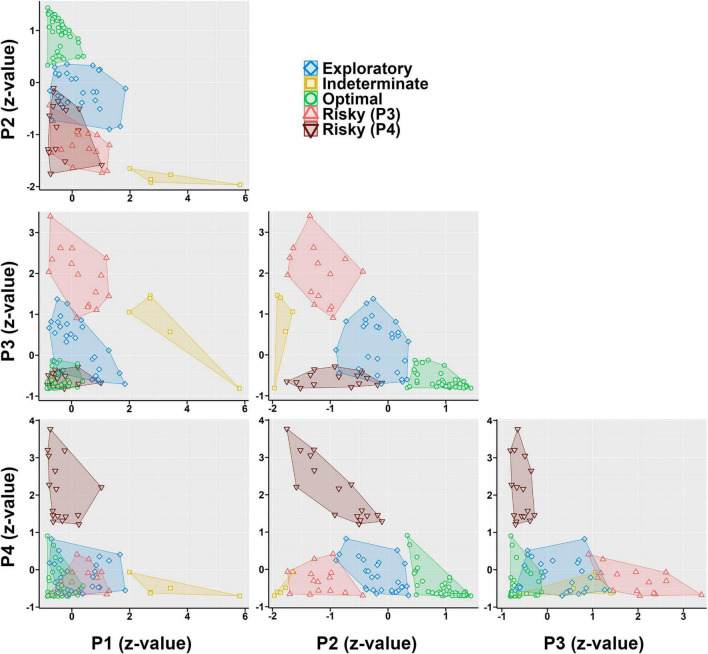
Normalized preference (z score) for a given option plotted against each other, with clusters superimposed on top. Points represent individual rats. An Optimal phenotype (green) can be seen as rats that have high P2 values and relatively low values of all others. An Exploratory phenotype (blue) can be seen with values across all options around 0 (average). Two risky phenotypes can be seen, one which highly prefers P3 (light red), and another which prefers P4 (dark red). Finally, a small cluster of indeterminate rats (yellow) can be seen with unique preference for P1.

**FIGURE 7:**
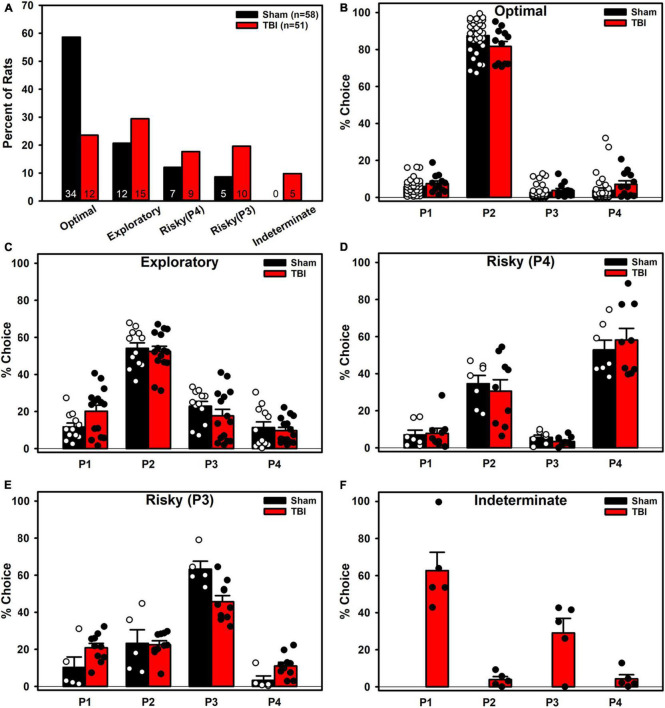
Phenotypes broken down by Injury. **(A)** TBI rats were significantly less likely to be in the Optimal phenotype and instead were increased across the other phenotypes. Only TBI rats were classified into the Indeterminate phenotype. **(B)** TBI was significantly different than Sham (*p* = 0.018) in the Optimal phenotype, with lower P2 and higher P4 choice. **(C)** TBI was not significantly different than Sham but had high variability in the Exploratory phenotype. **(D)** TBI was not significantly different than Sham but had high variance in the Risky (P4) phenotype as well. **(E)** TBI was significantly different than Sham (*p* = 0.001), with higher P1 and lower P3 preference in the Risky (P3) phenotype. **(F)** Only TBI animals were present in the Indeterminate phenotype.

The clusters that emerged represented five choice phenotypes (Figure 6): An Optimal (strong P2 preference), an Exploratory (moderate P2 preference), two Risky (a P3-preferring and a P4-preferring), and an Indeterminate (P1 + P3 preference). When these clusters were examined by group, a Fisher’s Exact Test revealed a significantly uneven distribution of cluster membership (*p* = 0.001; Figure 7A). TBI animals were less likely to be in the Optimal phenotype and displayed small increases in the remaining phenotypes. TBI animals were the only ones to demonstrate the Indeterminate phenotype.

For each cluster, an ANOVA (Pct Choice ∼ Injury * Choice Option) was conducted to see if groups differed despite being grouped together in the k-means process (Figures 7B–F). TBI were significantly different in the Optimal (Injury*Choice: *p* = 0.018) and Risky (P3) cluster (Injury*Choice: *p* = 0.001). To obtain a coarse measure of differences in variance, the standard deviations were calculated for each choice option for both groups and then summed for each phenotype to provide a qualitative comparison of variance. TBI had higher variance in the Risky (P4) cluster (48.11 vs. 35.76) and Exploratory cluster (43.77 vs. 36.73), less variance in the Risky (P3) cluster (31.34 vs. 43.94), and similar variance in the Optimal cluster (24.13 vs. 24.67).

### Supplemental Analyses

There were no differences on overall behavior between craniectomy and intact shams (Supplementary Figure 2), and so molar/molecular/atheoretical analyses were not applied to this group. Unilateral parietal TBI significantly decreased molar sensitivities, but increased tendency to stay with those options relative to bilateral frontal TBI (Supplementary Figures 3–5). With regard to the subset where pre- and post-injury performance was available, TBI significantly decreased molar and molecular sensitivity (Supplementary Figures 6–8).

## Discussion

To understand how to treat the psychiatric-like symptoms which stem from TBI, more research is needed regarding changes in behaviors which underlie these conditions. In the current report, we pooled data collected over multiple studies to better explore these fundamental changes. This enabled evaluation of the impact of individual variability and analyses of conditional data (e.g., a switch in choice after a loss). Rats performed more poorly on the RGT after TBI and choices tend to be allocated away from optimal options and toward both safer, suboptimal choices and riskier choices, suggesting a reduced sensitivity to the outcomes of choices (Figure 1). Notably, this could be explained by reduced molar sensitivity (i.e., sensitivity to overall contingencies) or by changes in molecular sensitivity (i.e., immediate outcomes: a “win” or “loss”).

An evaluation of the molar perspective of behavior was carried out in Experiment 1. There were substantial, statistically significant reductions in sensitivity to reinforcement and increased bias toward lower reinforcement rates in TBI rats (Figure 3). On the surface, this could potentially explain how TBI changes decision-making. However, a closer consideration of the data reveals significant problems with this interpretation. From a pure optimization standpoint, a task such as this theoretically should generate exclusive preference of the P2 option to maximize reinforcement. However, this is clearly not the case for almost any rats. This means that theoretically, the matching law should poorly describe the data. Indeed, at the subject level, we see this is the case for many subjects (Figures 2, 3). True matching behavior would result in a sensitivity (slope) of 1, and a bias (intercept) of 0. However, even in sham rats, there are a number with negative sensitivity and bias, otherwise understood as a preference for lower rates of reinforcement. Despite this problem, a shift in overall sensitivity to outcomes cannot be ruled out as this task was not explicitly designed to test this hypothesis. Rather, data of convenience were used to provide a rough evaluation. Indeed, studies explicitly controlled to examine matching under similar conditions find that adjustment of choice probabilities across blocks will generate matching in a 3-alternative probabilistic task (Kangas et al., 2009). Moreover, in patients with TBI, when a similar adjusting probability procedure is used, patients displayed reduced sensitivity to changes and some tended to overestimate their own performance in self-report (Schlund and Pace, 2000). Thus, while the matching law provides some marginal utility to describe behavior on the RGT, it does not capture the full range of individual subjects nor the depth of changes in choice behavior after TBI.

Because the RGT, with its fixed contingencies, is not well-suited for evaluating matching performance, an alternative might be to evaluate molecular sensitivity to immediate outcomes (i.e., “wins” and “losses”) on the task. This molecular perspective could potentially explain post-injury changes in which reduced sensitivity to negative outcomes (“losses”) or increased sensitivity to positive and/or large magnitude outcomes (“wins”) may have an outsized influence on behavioral impairment. Patient data suggest that TBI leads to less sensitivity to negative outcomes because they display less reactivity to fearful stimuli alongside poor IGT performance (Visser-Keizer et al., 2016). Despite this, in the current data, we did not find any significant differences in sensitivity to wins vs. losses on the RGT. TBI rats were significantly changed overall in their tendency to stay with a given option (Figure 4), but both TBI and sham had downward shifts in probability following losses. Further, this was not uniquely affected by the choice options with more frequent wins or losses. Interestingly, we again observed drastic individual differences (Figure 5), with both sham and TBI rats displaying a range from exclusive choice to almost absolute alternation amongst options. Thus, it seems that some level of differential sensitivity to immediate outcomes is not a driver of TBI-induced deficits on the RGT. Rather, the overall changes suggest further support for a more molar viewpoint as discussed above.

Neither the molar nor molecular approach fully explained choice behavior on the RGT. To determine if a theory-agnostic approach would provide more explanatory power, k-means clustering was performed on the full dataset. This generated five total choice phenotypes: Optimal, Exploratory, two Risky, and Indeterminate. There was minimal overlap between the pattern of choices for rats in any given phenotype and can be visualized by plotting each choice against each other (Figure 6), yielding distinct patterns which clearly segregate even optimal from exploratory rats. These phenotypes described choice behavior very well, and moreover, the changes in TBI animals were accounted for almost entirely by a reduction in the Optimal phenotype (Figure 7). However, clustering results should always be approached with some level of caution. K-means and similar algorithms are designed to maximize variance accounted for, and so further explorations should be performed to determine if the phenotypes found here hold up across future studies or in other laboratories. Another consideration is that these phenotypes may merely recapitulate the matching data. Rats who were true matchers (i.e., sensitivity approximately 1) are likely those in the Exploratory phenotype, while those with the highest sensitivity and positive bias are the Optimizers, and those with negative matching or bias are likely the Risky rats. Still, these phenotypes illustrate that the matching data are less continuous than might be inferred from the aggregated plots and that distinct clusters of preference emerge on the RGT. Finally, these phenotypes open new avenues of investigation into the underlying neurobiology or behavioral drivers of such choice. For example, differences in phasic dopamine activity and/or dopamine receptor and transporter density may underly TBI-mediated cognitive deficits (Bales et al., 2009) as well as reactivity to conditioned stimuli (e.g., the choice hole) and primary reinforcers (e.g., the sucrose pellets) for intact rats (Singer et al., 2016).

The current data do not fully explain how choice behavior develops on a probabilistic task such as the RGT. However, they do inform our interpretation of how stable behavior is best described and how it is altered by a brain injury. A prior study using similar retrospective data (in intact rats) suggested a combination of immediate consequences and molar contingencies drove acquisition of behavior on this task using a model of reinforcement learning (Langdon et al., 2019). Specifically, it was suggested that reductions in loss sensitivity would allow for a riskier phenotype and this could be augmented by pairing complex audiovisual cues with riskier options. Interestingly, given the current data demonstrating large-scale shifts in phenotypes immediately following TBI, a lack of explicit changes in loss sensitivity, and a tendency toward molar-level insensitivity, the prior study may not fully explain the development of risky decisions. Unfortunately, these comparisons are somewhat limited by our current selection of only stable post-injury data (i.e., 4 + weeks post-injury). While this gave the ability to compare a large amount of data, it limited what could be interpreted about acquisition of this task in TBI rats. Ultimately, the current study highlights the need to experimentally manipulate these parameters so that we can dissociate molar from molecular tendencies in decision-making and evaluate whether the reported phenotypes have underlying neurobiological substrates. Further data collection in TBI animals both before and after the injury (*N* = 19 in current study; Supplementary Figures 6–8), exploration of differences in unilateral parietal and bilateral frontal TBI (*N* = 10 unilateral in current study; Supplementary Figures 3–5), and evaluation of effective therapeutics may also provide insights on the biobehavioral pathologies which drive maladaptive decision-making. Using data collected from such studies, we may be able to devise rehabilitative strategies to treat the devastating consequences of TBI.

## Data Availability Statement

Raw data are available via the TBI Open Data Commons (https://odc-tbi.org/), ODC-TBI accession ID 703 (Vonder Haar et al., 2022). Processing/analysis syntax may be requested by contacting the authors.

## Ethics Statement

The animal study was reviewed and approved by the West Virginia University IACUC.

## Author Contributions

CV, MF, VM, AR, and KM collected the data, drafted, and edited the manuscript. CV, MF, VM, and AR performed the analyses. All authors contributed to the article and approved the submitted version.

## Conflict of Interest

The authors declare that the research was conducted in the absence of any commercial or financial relationships that could be construed as a potential conflict of interest.

## Publisher’s Note

All claims expressed in this article are solely those of the authors and do not necessarily represent those of their affiliated organizations, or those of the publisher, the editors and the reviewers. Any product that may be evaluated in this article, or claim that may be made by its manufacturer, is not guaranteed or endorsed by the publisher.
