# Supplementary material for: Large-N Rat Data Enables Phenotyping of Risky Decision-Making: A Retrospective Analysis of Brain Injury on the Rodent Gambling Task
Source: Front Behav Neurosci. 2022 Apr 25;16:837654. doi: 10.3389/fnbeh.2022.837654 (PMC9084609; doi:10.3389/fnbeh.2022.837654)
Supplement: Supplementary file 1 [file Data_Sheet_1.docx]

**SUPPLEMENTAL MATERIAL**

**METHODS**

Comparisons were made amongst subgroups using the methods described in the main text. Specifically, a comparison of the overall performance of craniectomy versus intact shams was performed for each choice option, but no follow-ups because the effects were non-significant. A set of comparisons was made according to each approach (Exp 1, 2, 3) for unilateral parietal versus bilateral frontal TBI and to compare the within-subject effects of TBI.

**RESULTS**

**Craniectomy versus Intact Sham**

Results are shown in Figure S2. There were no significant differences in choice or choice by session for P1 (*F*_(1,56.21)_ = 1.47, *p* = 0.231; *F*_(1,56.87)_ = 0.22, *p* = 0.641), P2 (*F*_(1,55.97)_ = 0.60, *p* = 0.443; *F*_(1,56.46)_ = 1.13, *p* = 0.292), P3 (*F*_(1,56)_ = 3.02, *p* = 0.088; *F*_(1,56.40)_ = 2.94, *p* = 0.092), or P4 (*F*_(1,56)_ = 1.30, *p* = 0.259; *F*_(1,56.32)_ = 3.83, *p* = 0.055).

**Comparison of Unilateral versus Bilateral Injury**

For molar effects (Experiment 1; Figure S3), sensitivity to reinforcement was significantly lower in the unilateral TBI group (*t*_(48)_ = 3.13, *p* = 0.003) and bias toward risky options increased (*t*_(48)_ = 4.08, *p* < 0.001). However, the groups were equal in their poor fit of the matching law (*t*_(25.11)_ = 1.69, *p* = 0.103).

For molecular effects (Experiment 2; Figure S4), unilateral TBI increased the likelihood to stay with an option overall and across sessions (*F*_(1, 72.06)_ = 6.40, *p* = 0.005; *F*_(1, 833.63)_ = 19.11, *p* < 0.001). However, when analyzed by win/loss, there was no differential sensitivity, only the main effect of injury type (*F*_(1, 1714.03)_ = 0.38, *p* = 0.539; *F*_(1, 73.48)_ = 11.09, *p* = 0.001).

For atheoretical clustering (Experiment 3; Figure S5), there was no significant difference in the distributions (Fisher’s exact test: *p* = 0.434), however this test has relatively low power given the multiple phenotypes/conditions and low number of observations.

**Within-Subject Comparison of Pre- versus Post-Injury**

For molar effects (Experiment 1; Figure S6), sensitivity to reinforcement was significantly lower after injury (*t*_(18)_ = 2.57, *p* = 0.019), bias toward risky options increased (*t*_(18)_ = 2.47, *p* = 0.024), and fits of the matching law were reduced (*t*_(18)_ = 3.14, *p* = 0.006).

For molecular effects (Experiment 2; Figure S7), injury reduced the likelihood to stay with an option overall (*F*_(1, 496.08)_ = 9.08, *p* = 0.003). However, when analyzed by win/loss, there was no differential sensitivity, only the main effect of injury type (*F*_(1, 1009)_ = 0.41, *p* = 0.522; *F*_(1, 1009.1)_ = 10.88, *p* = 0.001).

For atheoretical clustering (Experiment 3; Figure S8), there was no significant difference in the distributions (Fisher’s exact test: *p* = 0. 282), however this test has relatively low power given the multiple phenotypes/conditions and low number of observations.


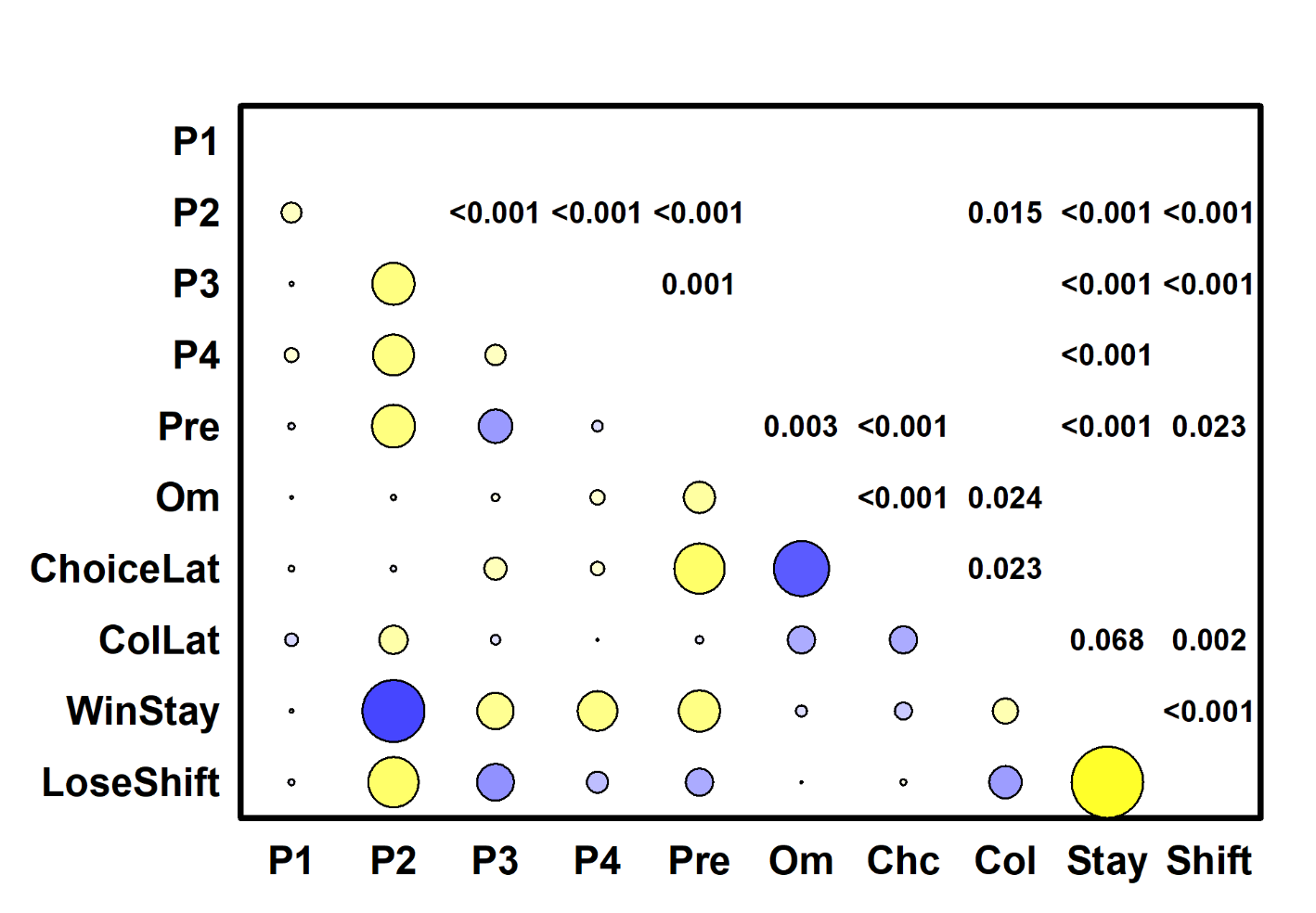


**Figure S1.** Correlations between various measures on the RGT. Measures that were fully- or semi-dependent on others were removed (e.g., total trials = choices+omissions+prematures). Correlation strength is indicated by size and color, with blue representing positive correlations, and yellow negative correlations. Corresponding p-values (Bonferroni-corrected) are provided for significant values.


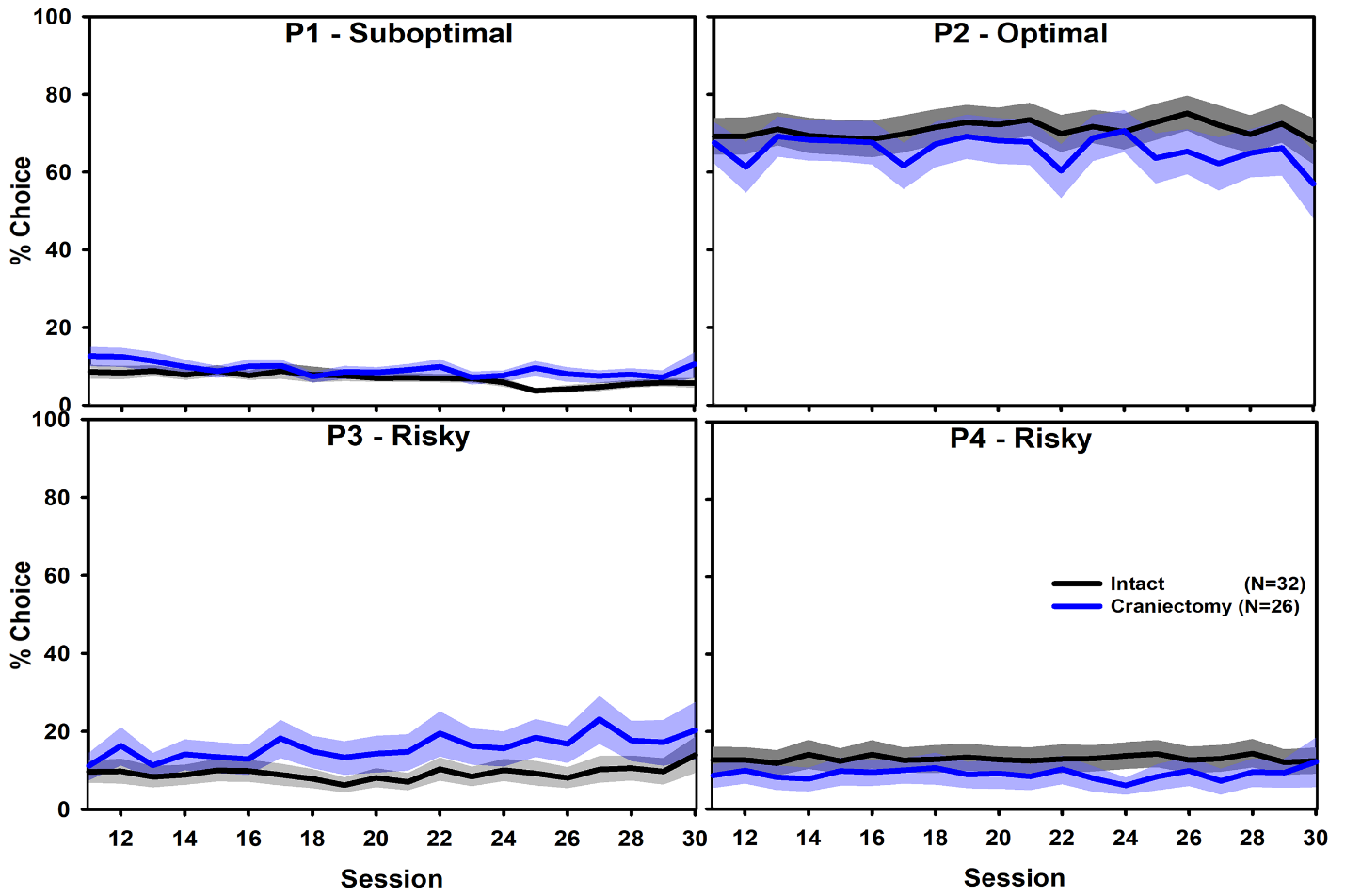


**Figure S2.** Performance on the RGT for craniectomy vs. intact shams. Lines represent mean and shaded band the SEM. There were no significant differences in choice for any of the options (lowest *p* = 0.088 for P3 option) or group by session interaction (lowest *p* = 0.055 for P4 option), despite the considerable power in the sample (N=56).


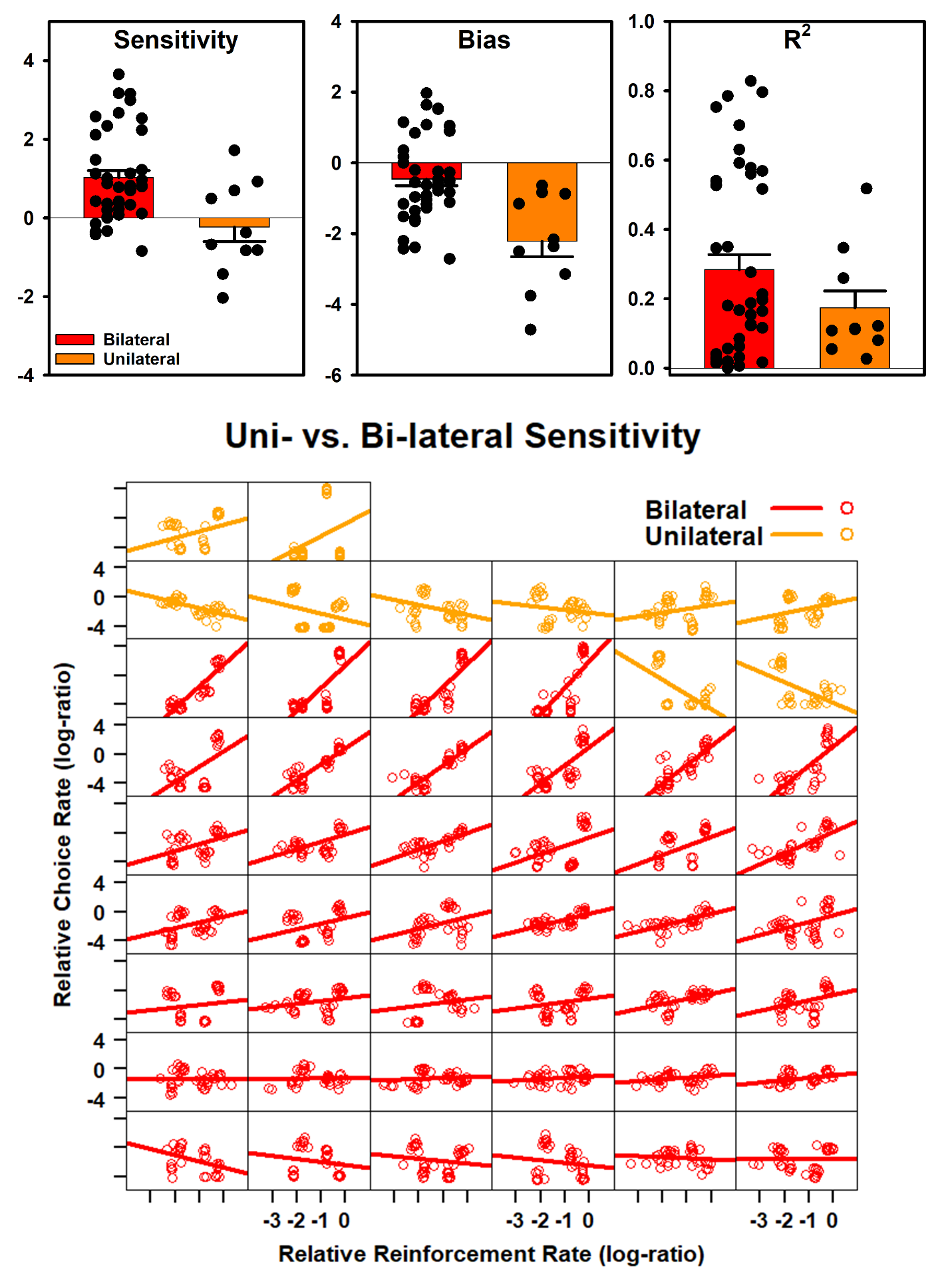


**Figure S3.** Comparison of bilateral and unilateral TBI on the matching law for molar sensitivities (Experiment 1). Unilateral injury surprisingly led to reduced sensitivity (*p* = 0.003), increased bias (*p* < 0.001), but similarly poor fits (*p* = 0.103) to the matching law. Individual subject fits showed the heterogeneity of this effect. However, results should be interpreted with caution due to the low N in the unilateral group.


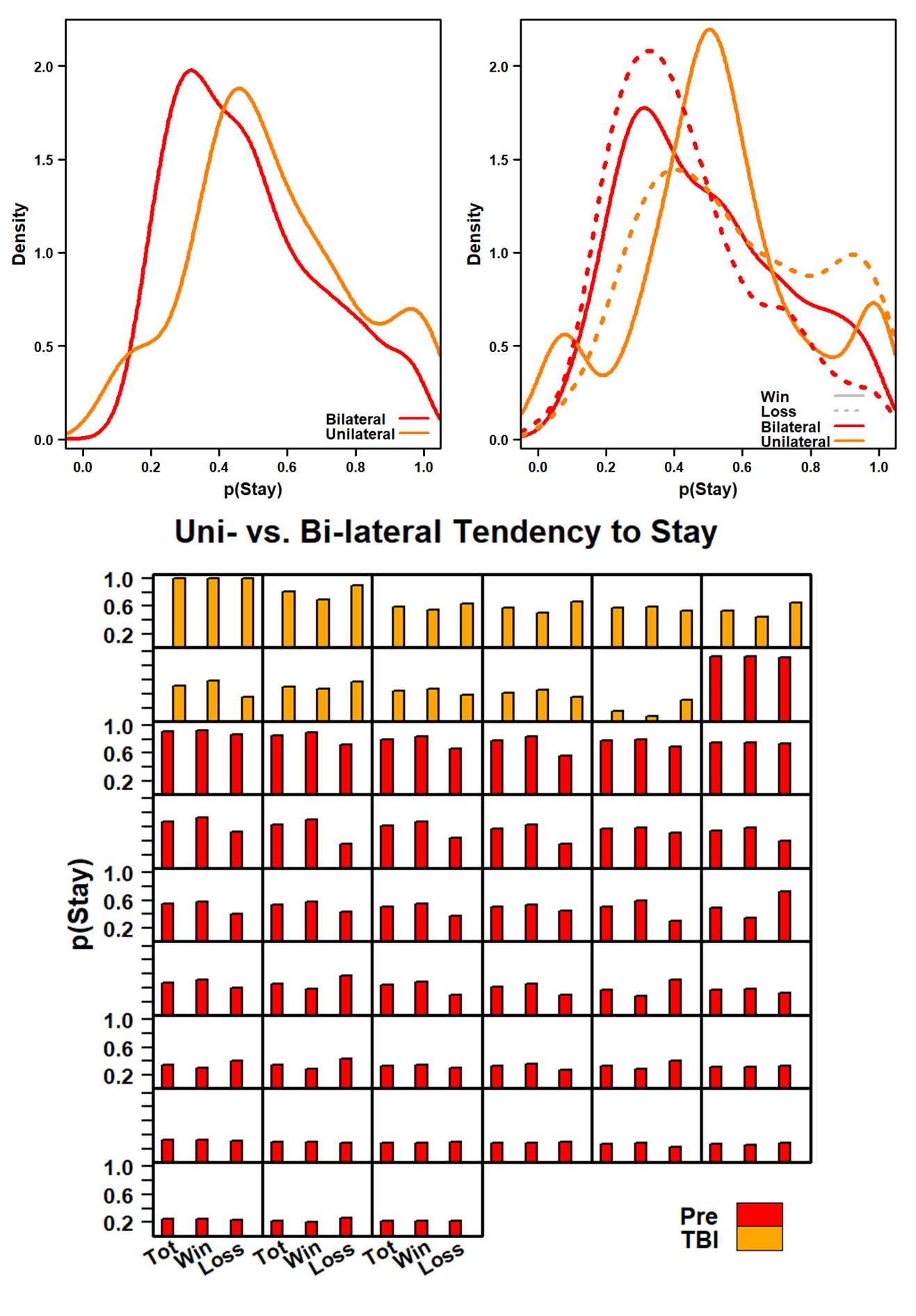


**Figure S4.** Comparison of bilateral and unilateral TBI on the tendency to switch after a given trial outcome for molecular sensitivities (Experiment 2). Overall, unilateral TBI rats were more likely to stay with an outcome, despite poorer choice profiles (*p* = 0.005), but were not significantly differential in their sensitivities to wins vs. losses (*p* = 0.539). Individual subject plots show the considerable heterogeneity in both groups.


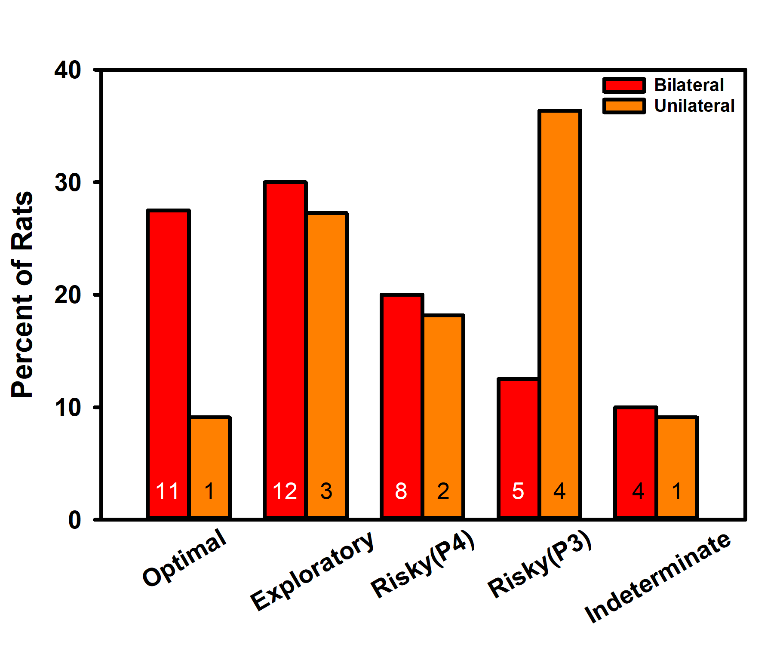


**Figure S5.** Phenotype assigned (Experiment 3) for bilateral vs. unilateral TBI. There was no significant difference in the distribution (*p* = 0.434), likely due to low power.


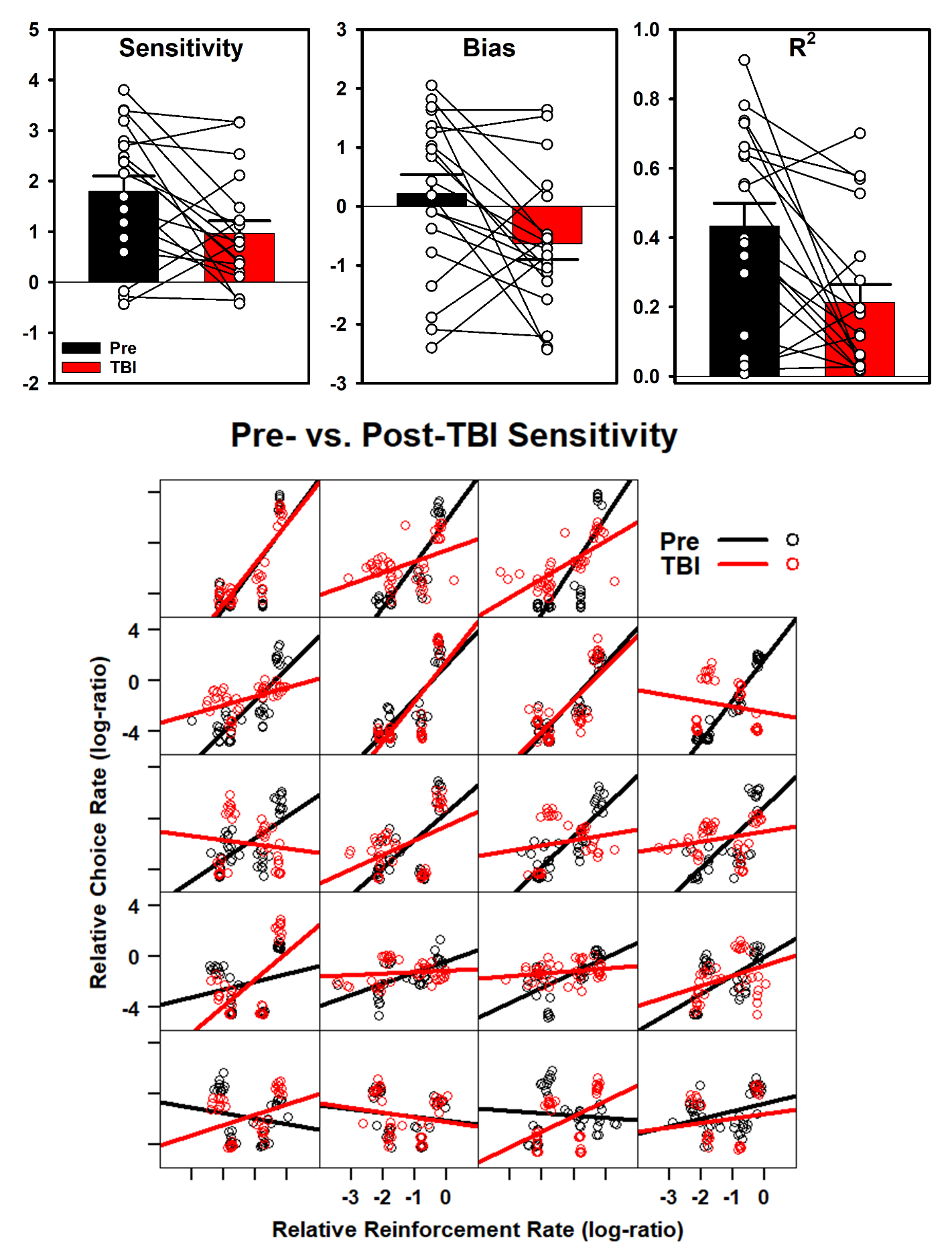


**Figure S6.**

Within-subject comparison of pre- and post-TBI on the matching law for molar sensitivities (Experiment 1). Injury reduced sensitivity (*p* = 0.019), increased bias (*p* = 0.024), and reduced fit (*p* = 0.006) to the matching law. Individual subject plots show variability in the response to injury.


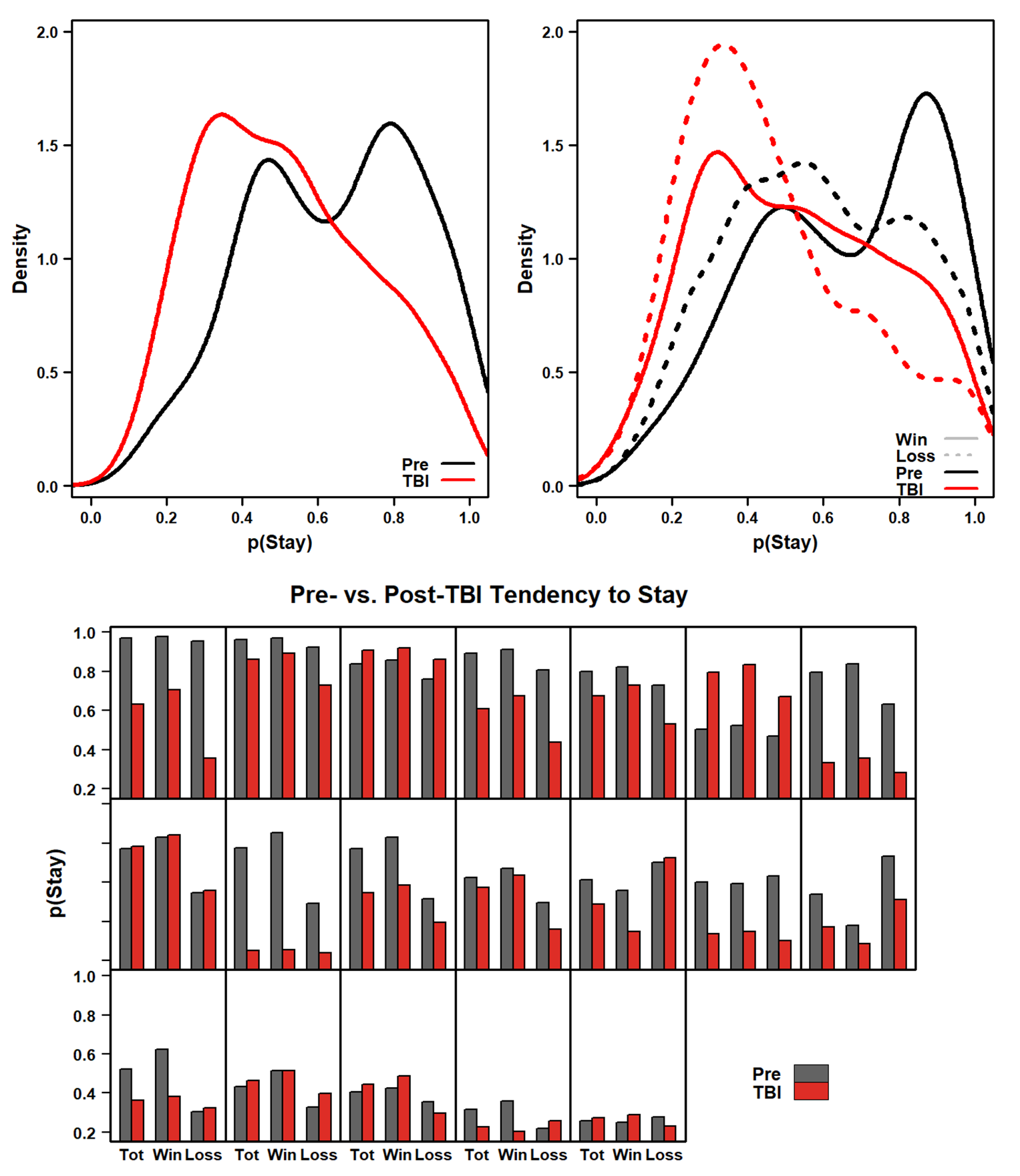


**Figure S7.**

Within-subject comparison of pre- and post-TBI on the tendency to switch after a given trial outcome for molecular sensitivities (Experiment 2). Overall, injury reduced rats likelihood to stay with an outcome (*p* = 0.003), but did not differentially affect likelihood between wins and losses (p = 0.522). Individual subject plots show variability in the response to injury.


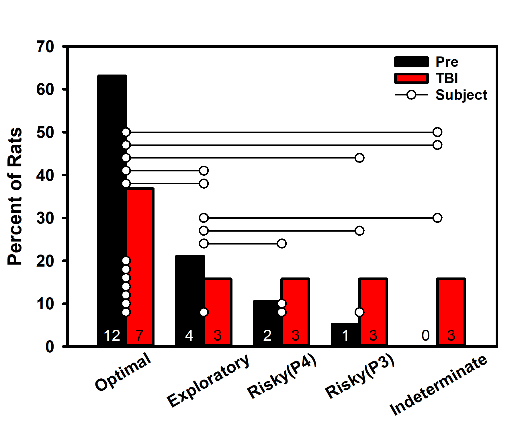


**Figure S8.** Within-subjects comparison of phenotype assigned (Experiment 3) during pre- vs. post-TBI. Dots indicate subjects and lines the trajectory/change in phenotype. Interestingly, TBI never led to improvements, but there were also a substantial number of subjects which were unaffected. There was no significant difference in the distribution (*p* = 0.282), likely due to low power.
